# Fluorescence microscopy imaging of mitochondrial metabolism in cancer cells

**DOI:** 10.3389/fonc.2023.1152553

**Published:** 2023-06-22

**Authors:** Monika Gooz, Eduardo N. Maldonado

**Affiliations:** ^1^ Department of Drug Discovery & Biomedical Sciences, Medical University of South Carolina, Charleston, SC, United States; ^2^ Hollings Cancer Center, Medical University of South Carolina, Charleston, SC, United States

**Keywords:** fluorescence microscopy, mitochondrial metabolism, NAD(P)H, FAD, mitochondrial membrane potential, ROS

## Abstract

Mitochondrial metabolism is an important contributor to cancer cell survival and proliferation that coexists with enhanced glycolytic activity. Measuring mitochondrial activity is useful to characterize cancer metabolism patterns, to identify metabolic vulnerabilities and to identify new drug targets. Optical imaging, especially fluorescent microscopy, is one of the most valuable tools for studying mitochondrial bioenergetics because it provides semiquantitative and quantitative readouts as well as spatiotemporal resolution of mitochondrial metabolism. This review aims to acquaint the reader with microscopy imaging techniques currently used to determine mitochondrial membrane potential (ΔΨm), nicotinamide adenine dinucleotide (NADH), ATP and reactive oxygen species (ROS) that are major readouts of mitochondrial metabolism. We describe features, advantages, and limitations of the most used fluorescence imaging modalities: widefield, confocal and multiphoton microscopy, and fluorescent lifetime imaging (FLIM). We also discus relevant aspects of image processing. We briefly describe the role and production of NADH, NADHP, flavins and various ROS including superoxide and hydrogen peroxide and discuss how these parameters can be analyzed by fluorescent microscopy. We also explain the importance, value, and limitations of label-free autofluorescence imaging of NAD(P)H and FAD. Practical hints for the use of fluorescent probes and newly developed sensors for imaging ΔΨm, ATP and ROS are described. Overall, we provide updated information about the use of microscopy to study cancer metabolism that will be of interest to all investigators regardless of their level of expertise in the field.

## 1 Introduction

Mitochondria and cytosolic glycolysis are both major drivers for survival and proliferation of cancer cells. The first link between mitochondrial function and cancer originated from the studies of Warburg in the early 1920’s who found that tumors produce more lactic acid than non-tumor cells. Warburg even proposed that damaged mitochondria caused cancer (1, 2). Although his theory was proven wrong, the findings about enhanced cytosolic glycolysis in cancer cells remained a central concept in cancer metabolism until present. Regardless of the relevance of glycolysis for proliferation, mitochondria are also essential for cancer cell homeostasis and to sustain tumor growth (3–5). Both enhanced glycolysis and partially suppressed mitochondrial metabolism characterize what today we call the Warburg phenotype (1, 2, 5–8).

Research in the last 20 years has shown that the Warburg effect is not restricted to cancer cells (9–11). Fibroblasts provide not only structural integrity but are involved in intercellular signaling and tissue homeostasis (12, 13). Skin fibroblasts activated in response to wound healing and inflammation, also referred to as myofibroblasts, consume more glucose and secrete more lactate than normal fibroblasts (11, 14). Like myofibroblasts, cancer associated fibroblasts found in almost all solid tumors display enhanced glycolysis, high levels of pyruvate, ketone bodies and lactate that are released and utilized as fuels to sustain mitochondrial metabolism in tumor cells. This phenomenon has been described as a Reverse Warburg Effect, in which epithelial cancer cells induce the normal stroma to become a wound-healing type that provides an energy rich environment that facilitates tumor growth (9, 10, 15). Thus, a comprehensive approach to the study of mitochondrial metabolism in cancer would greatly benefit from considering the role of mitochondria both in tumor cells and the supporting microenvironment (16).

**Figure 1:**
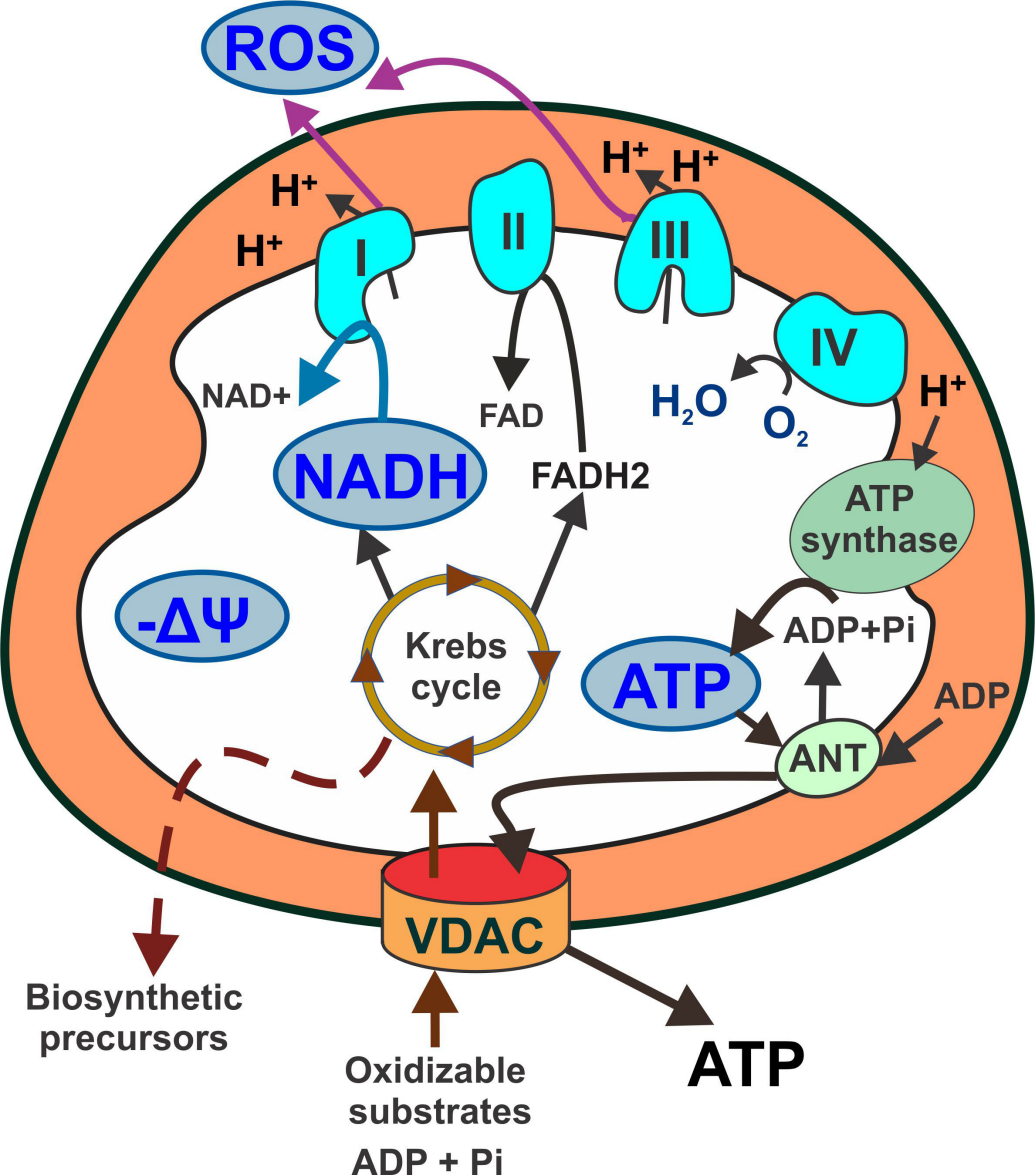
Schematic representation of mitochondrial metabolism highlighting main readouts used in fluorescence microscopy. Respiratory substrates (pyruvate, fatty acylCoA and certain amino acids) enter mitochondria through VDAC and fuel the Krebs cycle. NADH is the main electron donor for complex I. Electron flow through complexes II, III and IV is associated with proton pumping to the intermembrane space that generates a proton motive force used by the ATP synthase to produce ATP. ATP exchanges ADP for ATP. Proton accumulation in the intermembrane space creates a negative voltage (mitochondrial membrane potential) across the mitochondrial inner membrane. Respiratory complexes I and III are also major production sites of mitochondrial reactive oxygen species. Light blue shapes: major mitochondrial metabolic indicators. Orange: intermembrane space. ANT: adenine nucleotide translocator; ATP: adenosine triphosphate; FAD: Flavin adenine dinucleotide; NADH: nicotinamide adenine dinucleotide; ROS: reactive oxygen species; VDAC: voltage dependent anion channel; ΔΨ: mitochondrial membrane potential.

Mitochondrial metabolism is sustained by the oxidation of substrates that leads to the generation of ATP, reactive oxygen species (ROS), and biosynthetic precursors. Voltage dependent anion channels (VDAC) are the only gateway in the outer mitochondrial membrane for substrates, ADP and inorganic phosphate to enter mitochondria and for mitochondrial ATP to be released to the cytosol. Oxidation of pyruvate, fatty acyl CoAs, and certain amino acids in the Krebs cycle generates NADH and FADH_2_ in the mitochondrial matrix. NADH is the main electron donor to the electron transport chain (ETC). The flow of electrons through the ETC is associated with H^+^ pumping to the intermembrane space (IMS, orange in **Figure 1**) and ROS formation. Accumulation of H^+^ in the IMS generates a negative voltage in the mitochondrial matrix and a proton motive force used to synthesize ATP. The activity of the ETC coupled to ATP synthesis is called oxidative phosphorylation (Oxphos). The negative voltage in the matrix is the mitochondrial membrane potential (ΔΨm) (**Figure 1**). Oxidative metabolism refers to a state of mitochondria oxidizing substrates and producing ATP at full capacity.

The relative contribution of glycolysis and oxidative phosphorylation to cancer metabolism is commonly determined by measuring ATP generation. Ten to 90% of total cellular ATP in tumor cells is contributed by cytosolic glycolysis, the rest being generated through Oxphos. This fact points outs the critical role of mitochondrial oxidative metabolism in cancer (17, 18). Although for a long time enhanced aerobic glycolysis has been associated with tumor aggressiveness, evidence from *in vitro*, *in vivo*, and epidemiological studies showed a very heterogenous contribution of glycolysis and Oxphos to cancer metabolism. Moreover, in some aggressive tumors oxidative metabolism prevails over glycolysis (19–24).

Access to oxygen and nutrients, among other factors, influences the balance between glycolysis and Oxphos. Inadequate perfusion in areas of rapid cell proliferation creates hypoxia and decreases the supply of nutrients including glucose. However, the influence of hypoxia on oxidative metabolism depends on the duration and cell type. Prolonged hypoxia increases glycolysis in MCF-7 cells but not in HeLa cells although in both cell lines, OxPhos is the predominant source of ATP (25). Noticeably, in solid tumors the respiratory chain is still fully functional at oxygen levels as low as 0.5%, indicating that hypoxic tumor cells exposed to <2% oxygen still produce ATP by OxPhos (26–28). If pyruvate oxidation in the Krebs cycle decreases, tumor cells adapt to oxidize more glutamine as an energy source to sustain tumor growth both through glycolysis and OxPhos (29). A reciprocal dependence of mitochondrial metabolism and enhanced glycolysis has been shown in several cancer cell types under hypoxia and limitations in the availability of nutrients (3, 22, 23, 30–32). Nutrient availability induces a switch from aerobic glycolysis to OxPhos in lymphoma cells and breast cancer cell lines cultured in glucose-free media (33, 34). Under glucose and glutamine limiting conditions, tumor cells adapt to utilize other sources including lactate, methionine, arginine, cysteine, asparagine, leucine, acetate, and even lipids and proteins from the microenvironment (3, 33–37).

In recent years, the study of mitochondrial metabolism in cancer became a hot research topic both for basic biology and for translational studies involving drug development and response to other forms of cancer treatment (38–45). In this review, we will focus on the use of advanced confocal and widefield microscopy for research on cancer metabolism in live cells. We will provide a detailed analysis of the use, advantages, and disadvantages of different imaging modalities to image ΔΨm, NADH, FAD, ROS, and ATP as major indicators of mitochondrial metabolism. These mitochondrial metabolic indicators provide valuable addition to the current markers used in advanced imaging technologies for cancer diagnostics (46–49).

## 2 Imaging modalities for mitochondrial metabolism

The most used microscopy imaging modalities for analyzing mitochondrial metabolism in live cells rely on the use of fluorescent dyes or the fluorescence of naturally occurring molecules. In recent years, a growing number of fluorescent molecules, probes and biosensors available, together with the lowering costs of widefield and confocal microscopes favored a widespread use of this technology. Simultaneously, the very fast developments in optical engineering and computational capabilities resulted in newer and sophisticated imaging approaches and advanced image processing. Thus, it is now possible to quantify and analyze different types of images, difficult to image molecules and to handle large datasets. Imaging mitochondrial metabolism has become essential to study cancer biology and cancer bioenergetics. Moreover, quantitative imaging of mitochondrial metabolism could be a potential exciting approach to identify novel biomarkers that could help to distinguish between normal, precancerous or cancer tissue, to predict effectiveness of chemotherapy or even to predict the metastatic potential of certain types of cancer (50, 51)

### 2.1 Epifluorescence widefield microscopy

**Figure 2:**
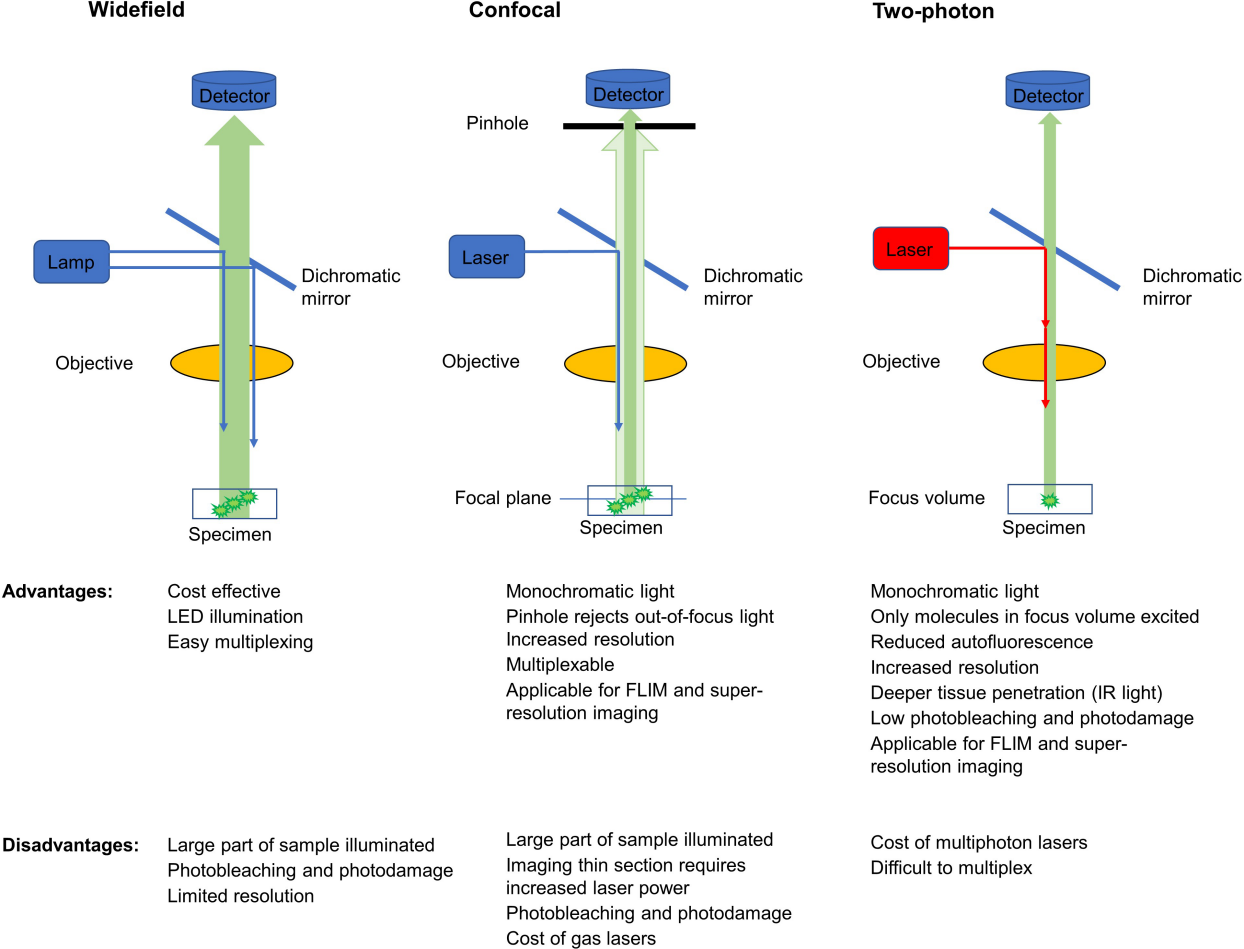
Comparison of signal detection methods in widefield, confocal and multiphoton microscopy (simplified schematics). Blue arrows show incoming single photon light and red show infrared multiphoton laser excitation. Green arrows show fluorescent light originating from the sample with darker green signal reaching the respective detectors. Excitation and emission filters are omitted.

It is one of the oldest fluorescent imaging modalities in which the specimen is illuminated using xenon and mercury arc lamps with wide wavelength spectrum. The development of image deconvolution algorithms and light-emitting diodes (LED) opened a new field in modern widefield microscopy. LED lights emit photons over a narrower bandwidth, do not produce heat, can be turned on/off quickly, and stay stable for longer periods of time compared to arc lamps. The incoming light in epifluorescence microscopy illuminates a large volume of the specimen (**Figure 2**). After passing through an emission/barrier filter, the signal is collected by a CCD camera or photomultiplier (PMT). The *advantage* of epifluorescence microscopy is the cost-effectiveness, and that LED illumination is less damaging to the sample than laser light. A major *disadvantage* in widefield microscopy is that increased intensity of the excitation light increases both the specific signal and the background due to light scattering, resulting in decreased contrast and resolution, and photobleaching. To overcome this problem, z-stacks (series of focal planes on the z axis) collected during image acquisition are then processed using image deconvolution software. Deconvolution is a computational method that allows restoration of the image based on the point spread function of the imaging system. The resulting image has increased contrast and resolution. Widefield microscopy is utilized successfully for both 2D (xy) and 3D (xyz) fluorescence microscopy of mitochondrial metabolic indicators including NAD(P)H and FAD (52) and ΔΨm (38). This technique can be especially suitable during drug discovery to measure ΔΨm, cellular ROS and ATP when subcellular resolution is not necessary.

### 2.2 Confocal microscopy

Laser-scanning confocal microscopy (LSM), use lasers (recently, the most used are diode lasers) to generate bright and monochromatic light. Both in widefield and LSM the energy of a single photon is utilized to excite endogenous or exogenous fluorophores in specimens ranging from 10 to 50 µm of thickness. Recently, it has been possible to reach about 200 µm depth during 3D imaging. Similar to widefield microscopy, incoming light illuminates a large volume of the specimen which is a *disadvantage* of the technique. However, in confocal microscopy a variable aperture called pinhole positioned in front of the detector rejects the out-of-focus light coming from below or above the focal plane. The smaller the pinhole, the less emitted light collected and the thinner the slice from which the fluorescent signal is collected. The correct choice of the pinhole size allows to decrease the background signal and to increase the signal-to-noise ratio. However, pinhole size must be carefully selected based on the objective used and signal intensity. Closing the pinhole too much decreases the specific signal and imaging requires higher laser power to excite the fluorophores which can eventually lead to photobleaching. To increase contrast and resolution, it is advisable to take z-stacks of the sample that are later processed by image deconvolution. The *main advantages* of confocal microscopy compared to epifluorescence widefield microscopy are the increased signal-to-noise ratio, higher resolution, and the capability of selecting a specific focal plane of the specimen to be imaged.

Widefield and LSM evolved over the years from microscopes used to image one or few specimens to newer high content imaging systems. High content imaging allows the simultaneous screening of mitochondrial metabolism in a large number of samples using 96 well-plates or 384 well-plates. This approach is very useful for screening chemical libraries or to identify pathway inhibitors/activators among other uses. The goal in these studies is to analyze signals at the cellular or subcellular level for which resolution of 20x-40x objectives are usually suitable (38). Metabolic parameters are usually expressed as relative fluorescence intensity per cell with or without deconvolution after background subtraction. Cell number is determined after segmentation using non-fluorescent cell counting (brightfield techniques), nuclei labeling with a fluorescent live nuclear dye like Hoechst, or by fluorescent plasma membrane staining (*e.g.*, CellTracker Green) (38). Quantitative results can also be expressed normalizing fluorescence intensity per total mitochondrial mass, as described below.

Overall, single-photon fluorescence is extensively used to measure fluorescent dyes and sensors to determine mitochondrial membrane potential, ATP and ROS production among others. However, imaging NAD(P)H and FAD autofluorescence that are sensitive to photobleaching requires a gentler approach (see below and **Table 1**).

**Table 1:** Imaging conditions for autofluorescent molecules.

Fluorophore	Excitation (nm)	Emission (nm)	Lifetime (ns)	Method	References
NAD(P)H	720	460/50		2P microscopy, *Zeiss 880*	(39)
NAD(P)H, flavins	330 and 440	multispectral		Multispectral digital colposcope	(50)
NAD(P)H, FAD	810	475 540		2P microscopy, *Zeiss META detector*	(51)
NAD(P)H, FAD Redox ratio	360/40 470/40	455/50 520/40		Widefield *DeltaVision* MATLAB pixel by pixel FAD/(NADH+FAD)	(52)
NAD(P)H	760	300-500 and 500-640	2.79/2.52 2.08/1.33	FLIM (2P) TCSPC *Dermainspect*	(53)
NAD(P)H, flavins	365 and 436	465/25 530/30		Widefield	(54)
NADH, NADPH	700	460/25	1.5 ± 0.2 4.4 ± 0.2	FLIM	(55)
NAD(P)H	351	460/25		Confocal LSM	(55)
NAD(P)H, Flavin	750 900	410-490 and 510-650 510-650		2P microscopy and spectroscopy	(56)
NAD(P)H, FAD	360 454	455 505-550		Widefield Confocal	(57)
NADH, FAD Redox ratio	800 890	<490	2.35 2.05	2P FLIM TCSPC FAD/NADH pixel-by-pixel intensity by *Image J*	(58)
NAD(P)H, FAD Redox ratio	750 890	400-480 500-600	1.3 1.4	2P FLIM *(Bruker)* TCSPC NAD(P)H/FAD	(59)
NAD(P)H FAD Redox ratio	750 900	1.2-2.6	0.47/2.85	2P FLIM Zeiss 880 TCSPC FAD/NADH *Image J*	(60)
NAD(P)H, FAD Redox ratio	750 890	440/80 550/100	1-1.5 0.3-1.3	2P FLIM *Bruker* TCSPC NAD(P)H/FAD	(61)
NAD(P)H, FAD Redox ratio	385/15 450-488	432/18 515/15		Widefield FAD/(NAD(P)H+FAD)	(62)
NAD(P)H, FAD Redox ratio	820 910	<490 629/56		2P microscopy NAD(P)H/FAD Pixel-by-pixel, *MATLAB*	(63)
NAD(P)H, FAD Redox ratio	755 860	460/20 525/25		2P microscopy *Leica SP2* FAD/(NAD(P)H+FAD) *MATLAB*	(64)
NAD(P)H, FAD	780 780	447/60 496<		2P *custom-built*	(65)
NAD(P)H, FAD Redox ratio	375 473	469/35 520/35		Endoscopic imaging *Thorlabs* FAD/(FAD+NAD(P)H)	(66)
NAD(P)H	375	445/40		FLIM needle optical biopsy TCSPC	(67)

### 2.3 Multiphoton excitation microscopy

In multiphoton microscopy, endogenous or exogenous fluorophores are excited by two (or three) photons (68). In two-photon microscopy, pulsed lasers are usually employed where in high photon flux molecules absorb two photons of twice the wavelength of one-photon excitation (i.e., half the photon energy) simultaneously (68). Pulsed excitation in two-photon (infrared) microscopy allows the fluorophores to “relax” between pulses and therefore reduces potential photo-induced stress or damage (e.g., ROS production) as compared with one-photon UV illumination, which may trigger cytotoxicity (69, 70). Since two photons needed to be absorbed simultaneously by the fluorophore, only molecules localized in the single focal point of the laser will be excited (**Figure 2**.). Because fluorescence is only generated in the focal point, the emitted light can be then collected without the need of a pinhole aperture (68, 71).

While the *disadvantage* of multiphoton systems compared to single photon systems is the higher costs, the *main advantages* are: a) Reduced autofluorescence because less light is absorbed by cells and tissues; b) Reduced light scattering; c) Reduced photodamage; and d) Deeper tissue penetration by infrared (IR) light. Since this technique shifts stimulation of endogenous NAD(P)H fluorescence from the UV wavelength (~340-360 nm) to IR (~720-800 nm), less DNA damage occurs in the nucleus and mitochondria (72). Because of these advantages multiphoton microscopy is a better approach to image endogenous fluorescence of NAD(P)H and FAD compared with single-photon microscopy.

### 2.4 Fluorescence lifetime imaging microscopy

Fluorescence-lifetime is the time a molecule spends on average in the first excited electronic state before returning to the ground electronic state. The fluorescence-lifetime decay of the fluorophore population in the sample can be spatially recorded as an image and used to discriminate between target molecules. The *advantage* of FLIM is that it is usually not affected by the concentration of the fluorophore and by photobleaching (73). FLIM is also used to image Förster resonance energy transfer (FRET), a process in which the emission spectrum of the so-called “donor” fluorophore overlaps with the excitation spectrum of the “acceptor” fluorophore. If these fluorophores are in close proximity, during stimulation of the donor molecule, (λ_ExDonor_) energy transfer occurs to the acceptor molecule with concomitant decrease in the donor’s emitted fluorescence (λ_EmDonor_) and increase in the acceptor’s emitted fluorescence (λ_EmAcceptor)_). Intermolecular FRET is used to study protein-protein interactions (74), whereas intramolecular FRET is widely used in fluorescent probes to follow changes in the conformation of biosensors upon binding of the molecule of interest. FRET-based sensors are utilized for example for real- time measurement of mitochondrial and cytosolic ATP levels, both important determinants of mitochondrial activity and cellular energy production (75, 76) (see 3.4. for details). The *disadvantage* of FLIM is that the special microscopes and complex image analysis makes this technology more expensive and difficult to handle compared to regular confocal microscopes (77). In addition, FLIM imaging is slower than regular confocal microscopy. Nonetheless, FLIM is being increasingly used to image free and protein bound forms of NAD(P)H or FAD and to calculate redox state in live cells (for references see **Table 1**).

There is currently a high interest in establishing if there is a relationship between cancer heterogeneity, cancer progression and NAD(P)H/FAD signal intensity, and to validate these autofluorescent metabolites as cancer metabolic biomarkers (53). Since FLIM can allow us to better identify molecules and interaction with other molecules than traditional confocal techniques, this imaging modality can provide valuable additional data in the hand of expert users.

## 3 Applications of imaging to the study of cancer metabolism

### 3.1 Endogenous fluorophores and natural biomarkers

Fluorescence imaging depends on the stimulation of a fluorogenic signal by light. Naturally occurring fluorescent (autofluorescent) molecules in cells and tissues can be imaged without the addition of engineered fluorescent probes or dyes. These include the structural proteins collagen and elastin; amino acids with aromatic rings like tryptophan, tyrosine and phenylalanine; and the pigments melanin, keratin, and lipofuscin (78–80). Most importantly, metabolic co-factors like nicotinamide adenine dinucleotide (NADH) and flavins are utilized in autofluorescence imaging to study cellular redox state (**Table 1**).

Previously, autofluorescence was regarded as nothing more than a background signal (81). The fluorescence signal originating from reduced pyridine nucleotide was discovered by Warburg (82) and its cellular presence was investigated using near UV spectrofluorometry by Duysens (83). Seminal studies on localization of blue autofluorescence to mitochondria was conducted by Chance and Baltscheffsky (84). An exciting publication of Chance in 1962 described the emission spectra and energy state of cells and mitochondria, and also the first fluorescence microscopy recordings of redox state and mitochondrial autofluorescence in cells (85). In the same work, the author also speculated how fluorescence microscopy could be used to study “dynamics of metabolism within the cell”.

#### 3.1.1 NAD^+^/NADH

Nicotinamide adenine dinucleotide (NAD**^+^**) and its reduced form NADH, are made up of an adenine nucleobase and nicotinamide connected through phosphate groups. They are co-factors of numerous enzymes, mostly dehydrogenases or reductases. The primary role of NADH is to deliver electrons from one reaction to another. The concentration of NADH is highest in the mitochondria, compared to the cytosolic content and NADH concentrations in other organelles (86, 87). Glycolytic NADH is “transported” into the mitochondria through the malate-aspartate shuttle to reduce mitochondrial NAD^+^ to NADH. However, most of the mitochondrial NADH originates from beta oxidation of fatty acids and pyruvate by enzymes of the Krebs cycle in the mitochondrial matrix. Mitochondrial NADH is oxidized by Complex I (NADH:ubiquinone oxireductase) of the respiratory chain (**Figure 1**) for which NADH is the main electron donor.

Intracellular free NAD^+^/NADH ratio is an important readout of cellular metabolism. The balance between NAD+ and NADH regulates several enzymes including glyceraldehyde 3-phosphate dehydrogenase involved in glycolysis and the pyruvate dehydrogenase complex, which converts pyruvate to acetyl-CoA in the mitochondrial matrix. Recently, an NADH-binding pocket in voltage dependent anion channels (VDAC) has been proposed as a target to develop small molecules to regulate mitochondrial metabolism (39). NAD^+^ also participates in post-translational modification of proteins through ADP-ribosylation reaction (88) and protein deacetylation via sirtuins (89, 90). In addition to experimental techniques, computational models were also developed to study the role of cytosolic NADH/NAD+ ratio on the mitochondrial NADH/NAD^+^ ratio in various disease states, including ischemia reperfusion and in cancer (91).

**Figure 3:**
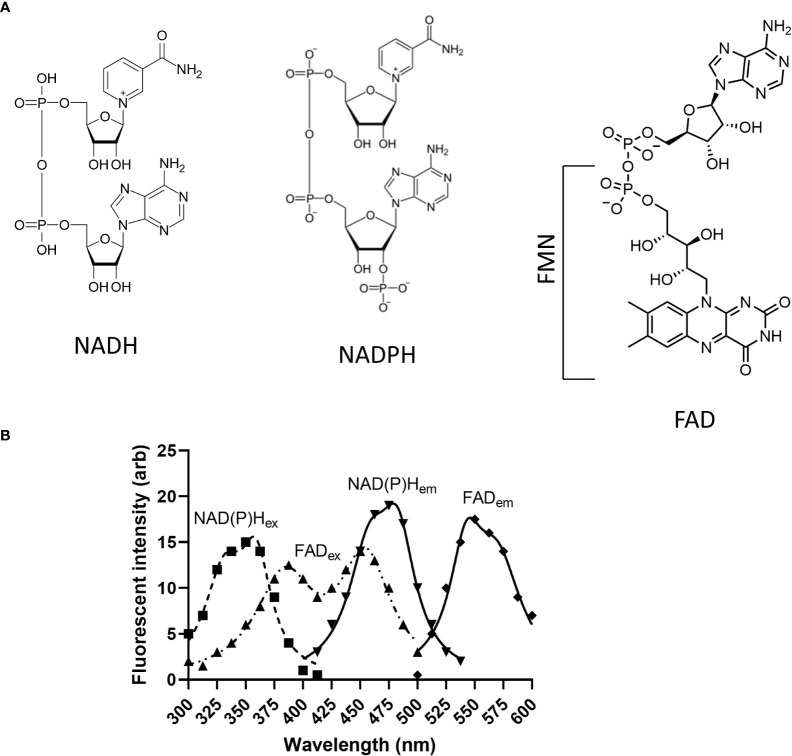
Structure and excitation/emission spectra of endogenous fluorophores discussed in this review. **(A)** Structures of NADH, NADPH, FMN and FAD; **(B)** NAD(P)H_ex_ and FAD_ex_ show excitation spectra of NAD(P)H and FAD, and NADP(H)_em_ and FAD_em_ depict emission spectra of NAD(P)H and FAD, respectively. NAD(P)H, Nicotinamide adenine dinucleotide (phosphate); FMN, Flavin mononucleotide; FAD, Flavin dinucleotide.

Spectroscopy analysis shows that both NAD^+^ and NADH have peak absorptions at 260 nm UV light. NADH has a second absorbance peak at ~340 nm which allows a ratiometric spectroscopic comparison of the oxidized and reduced forms (NAD^+^/NADH). NADH also fluoresces if excited at ~340 nm with maximal emission detected at ~440-465 nm depending on the solution and/or cell type utilized (54) and **Figure 3**. Interestingly, protein-bound NADH has blue-shifted emission spectra and higher fluorescence quantum yield than unbound NADH (92). This effect is caused by the unfolding of NADH after binding to proteins that prevents the adenine to quench the fluorescence of the nicotinamide ring. Accordingly, the fluorescence of NADH is lost when it is oxidized to NAD^+^ on complex I, allowing to follow the fate of the molecule and measure activity of complex I.

**Figure 4:**
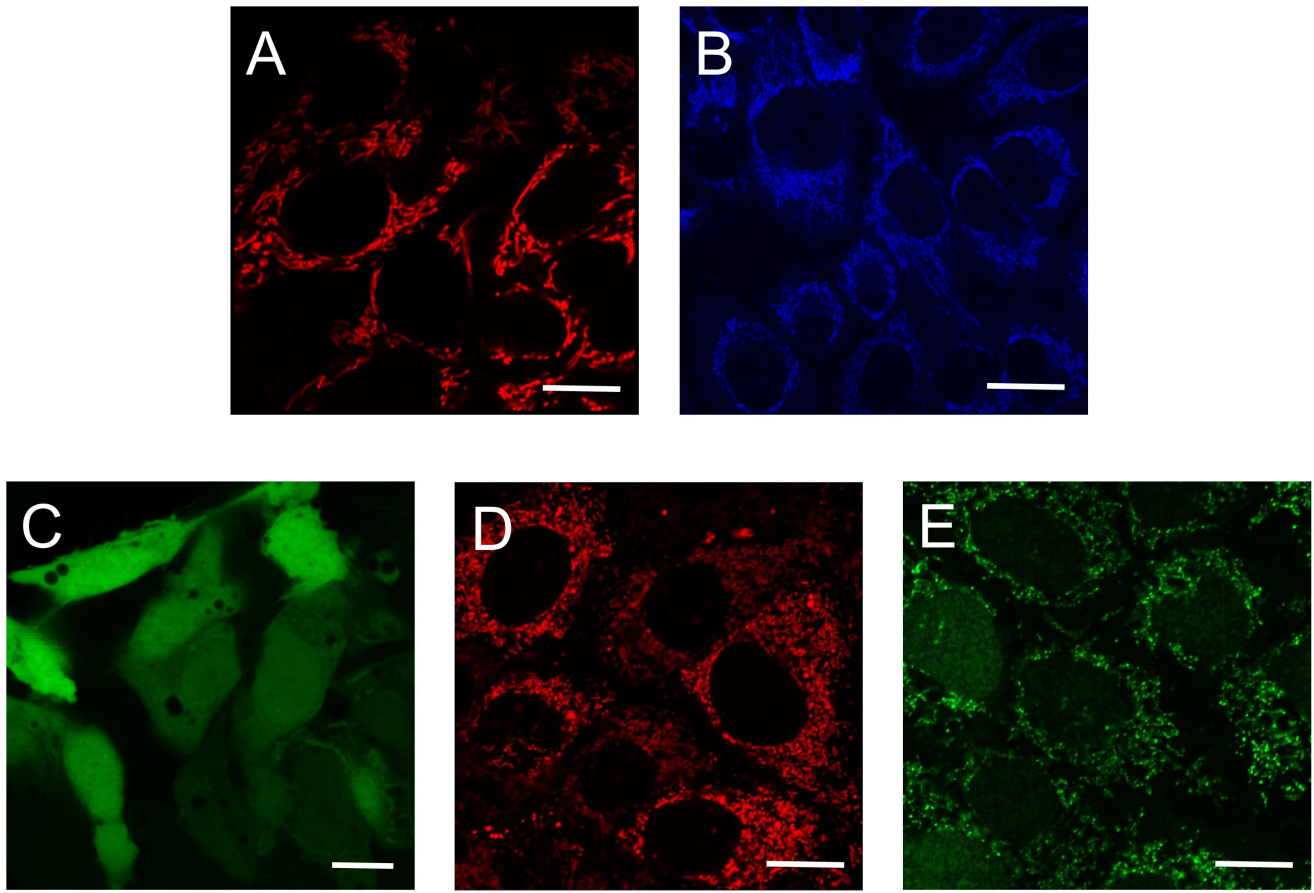
Use of autofluorescence and fluorescence dyes to determine mitochondrial metabolic activity. Single **(A, C, D, E)** and multiphoton confocal images **(B)** of human hepatocarcinoma cells. **(A)** TMRM-loaded mitochondria assess mitochondrial membrane potential; **(B)** Mitochondrial NADH autofluorescence; **(C)** CM-H_2_DCFDA fluorescence indicate cellular ROS; **(D)** MitoSox Red fluorescence is an indicator of mitochondrial superoxide; **(E)** CellRox Green fluorescence correlates with ROS formation. Scale bars: 10 μm. TMRM: tetramethylrhodamine methyl ester; CM-H_2_DCFDA: carboxymethyl 2’,7’-dichlorodihydrofluorescein diacetate.

To determine if autofluorescence originates from mitochondria, fluorescent non-potentiometric dyes (see below) are useful tools to label the mitochondrial network. Thus, NADH autofluorescence assessed by multiphoton microscopy can be imaged in parallel with single photon or multiphoton microscopy (**Figure 4**) to excite mitochondrial-targeted dyes. Subsequent colocalization analysis can determine if the autofluorescent signal originates in mitochondria. Another important consideration when imaging NADH autofluorescence is the use of positive and negative controls. Uncouplers like CCCP that create a futile cycle of oxidation in the respiratory chain together with oligomycin that inhibits the ATP synthase and Oxphos, results in an exhaustion of mitochondrial NADH with barely minimal production of ATP. By contrast, inhibition of the ETC leads to an accumulation of NADH. Specific inhibitors of complexes I and II lead to increased mitochondrial NADH. However, rotenone which is the most commonly used complex I inhibitor also disrupts microtubules (93–95). Microtubule destabilization creates an artifact that could influence NADH production by mechanisms unrelated to the inhibition of the ETC. We prefer to use the complex III inhibitor myxothiazol that prevent the flow of electrons to complex IV and maximizes the accumulation of NADH coming from the Krebs cycle (96) (**Figure 1**).

#### 3.1.2 NADP^+^/NADPH

Like NADH, reduced NADH phosphate (NADPH) is an electron carrier participating in cellular metabolism. In the cytosol, NADP is synthesized *de novo* from NAD^+^ by the cytosolic nicotinamide adenine dinucleotide kinase (cNADK) (97) and in the mitochondria by the mitochondrial NADK. NADP^+^ is reduced to NADPH in the pentose phosphate pathway, in the folate-mediated one carbon metabolism, by isocitrate dehydrogenase 1 and malic enzyme 1 in the cytosol. Mitochondrial NADPH is generated primarily from NADP and NADH by the nicotinamide nucleotide transhydrogenase (NNT) located in the inner mitochondrial membrane, and to less extent, by the citrate - α-ketoglutarate shuttle which reduces NADP to NADPH during the conversion of isocitrate to α-ketoglutarate and in the one-carbon and glutamate metabolism, and by malic enzyme 2/3. NADPH plays important roles in anabolic processes including fatty acid, amino acid and nucleotide synthesis (98). Importantly, NADPH serves as cofactor for the enzymes glutathione and thioredoxin reductase to maintain the glutathione peroxidase and the peroxiredoxin antioxidant system in the mitochondria. Maintenance of reduced glutathione levels (GSH) is essential to protect cells from aging and cell death induced by oxidative stress (99, 100). Further, in certain cancer cells, NADPH can promote generation of the oncometabolite D-2 hydroxyglutarate that favors cell survival (101). NADPH, therefore, is critical for proliferating cells. Interference of NADPH-dependent mechanisms is considered an important therapeutic target (102, 103).

An interesting bioinformatics analysis found that there are 352 enzymes in the liver (“hepatic NAD(P)ome”) which produce, consume NAD(P)H or depend on them as co-factors (104). There is increasing evidence showing that alteration in the concentration and subcellular distribution of NAD(P)H affects disease development and progression in aging, diabetes, Alzheimer’s disease and cancer. Although modulation of the biosynthesis of NAD(P) may seem a promising therapeutic area, the role of NAD(P) in various diseases still is not well characterized (105).

Since the absorption and emission spectra of NADH and NADPH are almost the same due to the nicotinamide moieties, it is appropriate to refer to the ~350 nm-excited autofluorescence as NAD(P)H (**Figure 3**). There is evidence that in mouse brain the level of mitochondrial NADPH autofluorescence is only 20% of NADH autofluorescence, and in some cases it appears insignificant compared to NADH fluorescence as confirmed by spectroscopy analysis (106). To distinguish between the contribution of the individual molecular species (NADH *versus* NADPH) to autofluorescence in cells of the cochlea, fluorescence lifetime imaging (FLIM) technique (see below) has been recently utilized (55, 107). As this method is not widely available, distinguishing between NADPH and NADH by autofluorescence imaging approaches is not practical. Commercially available colorimetric assays and HPLC (106) can be used to confirm NADH/NADPH ratio although these techniques lack the spatiotemporal resolution of the autofluorescence that allows the study of intracellular distribution of NADH and NADPH.

#### 3.1.3 NADH and NADPH biosensors

Genetically encoded NAD/NADH biosensors to quantitatively measure mitochondrial metabolic function are being developed to overcome the technical limitations to distinguish between NADH and NADPH autofluorescence (108–111). Biosensors reported are based on circularly permuted fluorescent proteins (cpFPs) fused to the NADH-binding domains of the bacterial Rex protein. Detection mode, dynamic changes, K_d_, pH sensitivity, emission and excitation wavelengths, brightness and type (intensiometric or ratiometric) of the biosensors Frex, FrexH, Peredox-mCherry, RexYFP, C3L194K and SoNar have been extensively reviewed (**Table 1** in (112)). Biosensors RexYFP, C3L194K and FREX have already been validated for mitochondrial application with dynamic changes of 50%, 300% and 800%, respectively (112). The utilization of the mitochondrial-targeted Frex sensor have allowed to measure cytoplasmic and mitochondrial free NADH levels (120 nM versus 30 μM) confirming data obtained from measuring intrinsic autofluorescence (87). The newest sensor, SoNar used in a high-throughput metabolic screening assay of anti-tumor compounds was able to determine that cytosolic NAD^+^/NADH ratio was between 100 and 900 in different cells. SoNar was also utilized for *in vivo* imaging of tumor xenograft in mice (113, 114).

NADP(H) biosensors recently developed include the Förster/fluorescent resonance energy transfer (FRET)- based Apollo-NADP^+^ (115). These sensors rely on the homodimerization of glucose-6-phosphate dehydrogenase in the presence of high levels of NADP^+^. Maintenance or depletion of cytoplasmic NADPH is an important tool to assess fitness of the cellular redox system during oxidative stress. Decreased NADPH level precedes the accumulation of hydrogen peroxide (H_2_O_2_), measured by the H_2_O_2_ sensor HyPer (115). Another group refurbished the NAD(H) sensor SoNar described above to the NADPH selective iNap to measure *in vivo* concentration of NADPH in HeLa cells and in a zebrafish wounding assay (116). Investigators showed similar NADPH levels in the cytosol as in the nucleus. Using the mitochondrial matrix-targeted iNap1 *versus* the cytosol targeted iNap3 sensor, mitochondrial NADPH level was ~37 μM compared to ~ 3 μM in the cytosol (116). Concomitant measurement of NADH and NADPH together with thiol and H_2_O_2_ sensor was also performed to image redox state in single cells opening an exciting possibility for high content multiparametric studies (117).

#### 3.1.4 Flavins and the redox ratio

Flavins are cofactors containing a tricyclic isoalloxazine ring that serve as a catalytic site. Mammalian cells use vitamin B2 (riboflavin) from food as a precursor to produce two important enzyme cofactors: flavin mononucleotide (FMN) and flavin adenine dinucleotide (FAD). Fully reduced (FMNH_2_, FADH_2_), fully oxidized (FMN, FAD), and an intermediate semiquinone state (FMNH^-^, FADH^-^) flavins accept or donate electrons in numerous redox reactions. Reduced flavins are nonfluorescent whereas fully oxidized flavins have absorption maxima at 370 and 440 nm with peak fluorescence at 520-530 nm (54) and **Figure 3**. Flavin autofluorescence is significantly quenched by binding to proteins. In certain cell types it is estimated that lipoamide dehydrogenase (LipDH) -bound FAD contributes up to 50% of the total flavoprotein-derived autofluorescence (117). Other flavoproteins in the mitochondria include NADH dehydrogenase in Complex I of the electron transport chain which contains FMN. This flavin is reduced when the mitochondrial electron carrier NADH is oxidized to NAD^+^. Other FAD-containing ubiquinone-reducing enzymes which feed electrons into the ETC include succinate dehydrogenase in Complex II, the electron transport flavoprotein-ubiquinone oxidoreductase (ETF dehydrogenase) and mitochondrial glycerol-3-phosphate dehydrogenase (118–120). Flavoproteins in the electron transport chain may contribute up to 25% of cellular autofluorescence (120).

The pool of mitochondrial NAD^+^/NADH influences the redox state of the flavin cofactors in LipDH and in the ETC-linked enzymes. Since only oxidized flavins (FMN, FAD) and reduced NADH are autofluorescent and they respond oppositely to changes in mitochondrial metabolic states, the ratio is also called “metabolic ratio”, which is an important indicator of cellular metabolism. A semantic issue should be considered when comparing publications because the same ratio is expressed differently, as NADH/FAD, FAD/NADH or as normalized optical redox ration of [FAD]/[FAD]+[NADH] or Fp/(NADH+Fp) (see reference examples below).

There is an increasing interest in label-free metabolic imaging including FAD. A PubMed search using “FAD autofluorescence imaging” yielded 2-4 publications per year between 2002 and 2006. However, this number increased to 32 in 2021. These metabolic parameters have been validated *in vitro*, for metabolic activity *ex vivo*, for *in vivo* experiments and also for non-invasive diagnostics studying metabolic activity of normal, precancerous and tumor cells (56–58).

NADH/FAD ratio can be used to distinguish between proliferating, quiescent or apoptotic cells by correlating the metabolic state with cell cycle status and cell proliferation. This approach could be particularly useful to study cancer heterogeneity, to differentiate cancer *versus* non-cancerous cell *in situ* and to evaluate the response to chemotherapy (59). Several recent publications have reported the use of combined imaging modalities to assess the NADH/FAD ratio. A detailed protocol to image NAD(P)H by epifluorescence microscopy and FAD by single photon confocal microscopy in live cells and tissue slices has been described (57). Multiphoton microscopy combined with FLIM was used to measure NADH and FAD lifetime (protein bound *versus* free) and the redox ratio to monitor metabolic changes during carcinogenesis *in vivo* in a model of oral cancer (58). A similar technique was used to correlate redox ratio (FAD/NAD(P)H) and ROS level with apoptosis using a FRET-based sensor of caspase-3 activity, mKate2-DEVD-iRFP in colorectal cancer cells (60). Multiphoton FLIM of autofluorescence was performed on patient-derived neuroendocrine cancer organoids and 2D cultures to follow treatment response. Optical metabolic imaging identified high cancer heterogeneity and confirmed that treatment response correlated with the redox ratio (61). Widefield (epifluorescence) imaging of optical redox ratio was used as a readout in a study that investigated the effect of combinational therapy on triple negative breast cancer cells (HCC1806 and MDA-MB-231). The decreased NADH and increased normalized optical redox ratio Fp/Fp+NADH after treatment correlated with cell growth inhibition indicating that optical redox imaging can be used as a biomarker of treatment efficiency (62). Label-free metabolic *intravital* imaging was used recently to study the redox ratio (in this publication NAD(P)H/FAD) in a triple-negative breast cancer model. It was shown that the ratio increased with tumor growth and decreased with anti-cancer treatment after anti-CD47 immunotherapy. The same technique was used to assess the metabolic activity of immune cells in the tumor environment (63).

Based on the advantages and disadvantages described in 2.3., 2.4. and above the authors recommend two-photon microscopy and FLIM for imaging NAD(P)H and FAD autofluorescence and the redox ratio (see also **Table 1** for details on excitation/emission wavelengths).

### 3.2 Mitochondrial membrane potential

Fluorescent dyes to determine ΔΨm, which is a relevant readout of mitochondrial function, also allow to visualize mitochondrial morphology and the mitochondrial network. Fluorescent dyes used to measure ΔΨm are lipophilic, cationic compounds that cross the mitochondrial outer and inner membranes and accumulate in the matrix based on its negative charge (121). ΔΨm results from a balance between the proton motive force, the mitochondrial pH gradient, and the proton leak from the intermembrane space to the matrix (**Figure 1**). Thus, increases or decreases in ΔΨm are good indicators of a functional integration between the activity of the ETC, the coupling of respiration to ATP synthesis and the proton leak. It is also a relevant parameter to measure together with production of ROS since the ETC is the main source of cellular ROS.

Maintenance of ΔΨm is a critical and highly dynamic process essential for mitochondrial function and cellular homeostasis (40, 122–125). ΔΨm is regulated at multiple levels, from the entry of respiratory substrates into the organelle, to the regulation of multiple transporters in the inner mitochondrial membrane or the modulation of the ETC and ATP synthase activities. Opening of the voltage dependent anion channel (VDAC) in the outer mitochondrial membrane, increased cytosolic Ca^2+^ or inhibition of the ATP synthase cause mitochondrial hyperpolarization (38, 39, 126) (127). VDAC closing by free tubulin that limit substrates entry into mitochondria of cancer cells or increased activity of uncoupling proteins are examples of decreased ΔΨm. Robust mitochondrial depolarization usually indicates mitochondrial dysfunction. Prolonged mitochondrial dysfunction eventually leads to cytochrome C release and cell death (128). Measurement of ΔΨm has been used as a readout to assess the effects of experimental drugs targeting mitochondria that induce metabolic stress through ROS production and to kill cancer cells (38, 127, 129).

A detailed review focused on discussing that potentiometric dyes can measure charge gradient ΔΨm between the intermembrane space and the mitochondrial matrix but not the proton motive force (130), also highlights many technical challenges. The amount of cationic dye accumulating in mitochondria is mostly determined by the negative voltage of the mitochondrial matrix. Because potentiometric dyes behave in a Nernstian fashion and plasma membrane potential (ΔΨp) is also negative, the accumulation of cationic dyes in mitochondria is influenced by both membrane potentials. Parallel measurements of ΔΨm and ΔΨp should be performed to determine if changes in the fluorescence of cationic dyes are caused by actual changes in ΔΨm and not to changes in ΔΨp (131–134).

An important factor to consider when analyzing ΔΨm is the intercellular and intracellular heterogeneity of ΔΨm. This phenomenon has been described in cancer cells using qualitative or semi-quantitatively methods (123, 130, 135, 136). However, a quantitative analysis of mitochondrial heterogeneity has only recently been published (22). Although it is a long-term known phenomenon, the molecular mechanisms underlying the differences in physiological and pathophysiological conditions remain undetermined. The high variability of ΔΨm poses a challenge for the metabolic characterization and should be taken into account in particular for the identification and analysis of cell subpopulations that are, very likely, metabolically heterogeneous. This should be considered not only for *intratumoral* differences but also between tumors of the same type, between primary and metastatic tumors and also for the same tumor at different times. Moreover, metabolically distinct cell populations are present even in cancer cell lines with the same genetic background. Thus, heterogeneity of ΔΨm should be included in the context of tumor heterogeneity and considered a factor to analyze when interpreting changes induced for genetic modifications or anticancer therapies that directly target or influence mitochondrial metabolism (22).

Because of the above-described considerations, it is of high importance to standardize dye loading concentration, loading time, temperature and utilize comparable methods to quantitatively analyze ΔΨm (22, 132, 137). While proper loading time is relatively easily to establish and loading temperature is mostly the temperature used for cell culture (for example 37 °C and 39 °C for human and mouse cells and cell lines, respectively), additional fine-tuning is needed to establish proper loading concentration of the fluorescent dyes to avoid mitochondrial toxicity, non-specific binding to other cellular structures, photodamage and dye aggregate artifacts.

#### 3.2.1 Fluorophores commonly used to determine mitochondrial membrane potential

Available mitochondrial membrane potentiometric dyes for fluorescent imaging include tetramethyl rhodamine methyl ester (TMRM, **Figure 3**) and ethyl ester (TMRE), Rhodamine 123 (Rho123), and the ratiometric dye 5,5′,6,6′-tetrachloro-1,1′,3,3′-tetraethylbenzimidazolyl-carbocyanine iodide (JC-1) (**Table 2**). TMRM is the most widely utilized. At low concentration (0.5 - 30 nM) or non-quenching mode for most cells, TMRM does not affect mitochondrial respiration (147) and easily loads various cell types. TMRM exhibits low photo-bleaching and can be imaged by confocal microscopy. For detecting rapid changes in ΔΨm, rhodamine 123 can be used at high, quenching concentration (130). A couple of excellent recent reviews discuss features and recommended utilization (including concentrations) of these dyes in cell culture (130, 132). Other mitochondrial dyes include MitoTracker probes that contain a thiol-reactive chloro-methyl group. These probes diffuse passively across membranes and accumulate in mitochondria. Some of the probes are selective to changes in ΔΨm like MitoTracker Red CMXRos but this dye is mostly utilized to image mitochondrial morphology (138, 139) rather than measure ΔΨm (148).

**Table 2:** Single photon excitation and emission wavelengths for fluorescent dyes to determine mitochondrial membrane potential, plasma membrane potential and reactive oxygen species.

Dye	Read-out	Excitation max (nm)	Emission max (nm)	Method	References
TMRM	ΔΨm	546/50 561	610< 576	Widefield Confocal	(121, 137) (127, 129, 131, 132, 138)
TMRE	ΔΨm	553	576	Widefield Confocal	(130, 131) (137)
Rhodamine 123	ΔΨm	507	529	Widefield	(130, 132, 137),
JC-1	ΔΨm	488	Ratio of 530 and 590 nm	Widefield	(130, 132),
MitoTracker Red CMX-ROS	mitochondria morphology, ΔΨm ()?	579	599	Confocal	(138, 139)
MitoTracker Deep Red FM	mitochondriamorphology	644	665	Confocal	(140)
MitoTracker Green FM	mitochondria morphology	490	516	Confocal	(141)
PMPI	ΔΨp	540	550	Widefield Confocal	(22, 133) (137)
MitoSox Red	superoxide (O_2_^-^)	396	610	Confocal	(127, 141),
MitoSox Green	superoxide (O_2_^-^)	488	510	Confocal	(142)
CM-H_2_DCFDA	cellular ROS	495	520	Confocal	(143)
CellRox Green	cellular ROS	485	520	Confocal	(144–146)
MitoTracker Orange CM-H_2_TMRos	mitochondrial ROS	554	576	Confocal	(143)
MitoTracker Red CM-H_2_XRos	mitochondrial ROS	579	599	Confocal	(143)

ΔΨm: mitochondrial membrane potential; ΔΨp: plasma membrane potential; ROS: reactive oxygen species.

See individual articles for detailed description of methods, wavelengths and filters used.

#### 3.2.2 Advantages of imaging mitochondrial membrane potential compared to flow cytometry and plate reader assays

ΔΨm can be determined not only by fluorescent microscopy but also by flow cytometry and plate reader assays. As mentioned above, loading potentiometric dyes into live cells requires optimization and careful consideration of loading conditions. Fluorescence microscopes with environmental control chambers (37 °C, 5% CO_2_ – or special buffered medium) are well suited for this task. However, even brief temperature changes (from an incubator to room temperature) during measurements can contribute to artifactual changes in ΔΨm. Therefore, although they are widely used, plate readers lacking environmental control or flow cytometry protocols when cells need to be stored on ice before measurement (149), do not provide an accurate measurement of ΔΨm. In addition, the surface to volume ratio (S/V) significantly influences dye loading. Trypsinization of attached cells to resuspend before measurement significantly changes their cellular structure and metabolic activity (for a recent review see (150)). Among the potentiometric dyes, JC-1 is one of the most commonly used for flow cytometry. JC-1 is a ratiometric dye that forms monomers or aggregates with different emission spectra (**Table 2**). Because aggregation is concentration-dependent and therefore, loading time dependent, any change in loading can lead to changes in intramitochondrial dye concentration making measurements inaccurate. JC-1 requires long loading times (~1.5 h). It has been shown that this dye is more suitable for detecting large changes if ΔΨm collapses (“yes/no” experiments) rather than more subtle modulations of ΔΨm. The carbocyanine DiOC_6_(3) is another ΔΨm indicator widely used in flow cytometry studies. The dye can only be employed at very low concentrations (<1 nm) due to its respiratory toxicity (151) and proved to be less reliable than JC-1 (152). Another important observation is that depending on the fluorescent dye and concentration, non-mitochondrial cellular structures can be labelled. This greatly influences data interpretation. Therefore, it would be useful to validate specific dye loading concentrations in fluorescence microscopy before flow cytometry and plate reader assays are performed.

In addition to the forementioned influence of uncontrolled temperature, flow cytometry and plate reader assays do not allow to study mitochondrial heterogeneity or to follow changes in mitochondrial metabolism over time. For example, this is a critical parameter to evaluate changes of mitochondrial metabolism induced to different drugs during drug development, to determine treatment efficiency and eventually to find mechanisms that cancer cells use to develop resistance to chemotherapy (136). Further, spatiotemporal distribution of signals cannot be studied due to lack of resolution. Due to the “yes/no” response of the dyes and cellular heterogeneity, fluorescence microscopy is more suited for quantitative analysis of drug responses.

#### 3.2.3 Newest developments: dyes with aggregation-induced emission and near infrared emission

Several dyes recently developed are not yet commercially available. The latest among them are dyes with aggregation-induced emission. In 2021, several new ΔΨm indicator dyes that do not accumulate in the mitochondria based on ΔΨm and do not require dye in the extracellular medium to image reversible ΔΨm dynamics, have been reported. SPIRIT RhoVR 1 is a mitochondrial targeted acetoxylmethyl ester originating from the Rhodamine Voltage Reporter, that freely penetrates into mitochondria (153). Once esterases remove the acetoxylmethyl group, it is trapped in the matrix, incorporates into the inner membrane, and serves as a reversible ΔΨm reporter. TPE-NT ([1-methyl-2-(4-(1,2,2-triphenylvinyl)styryl)-β-naphthothiazol-1-ium trifluorome-thanesulfonate), is a cationic fluorescence probe that accumulates in the mitochondrial inner membrane and its fluorescence is aggregation-induced (154). The dye loaded into LoVo human colon cancer cells was imaged by confocal microscopy using a 405 nm laser and emission at 625-725 nm (near infrared) which makes it easy to multiplex with other dyes. Interestingly, the dye’s response to CCCP seemed somewhat slow. Some of the aggregation induced dyes move out of mitochondria towards other organelles (lysosomes) after ΔΨm drops, and back to mitochondria again when ΔΨm increases again (154). This capability could potentially allow time-resolved quantitative imaging of ΔΨm but would make multiparametric imaging difficult. The development of new dyes underscores the importance of studying ΔΨm *in vivo*.

Since there are several fluorescent probes that were designed and characterized for single photon application, for imaging ΔΨm in live cells the authors recommend the use of widefield and confocal microscopy.

### 3.3 Reactive oxygen species

Reactive oxygen species are metabolic by-products implicated in cell signaling in physiological and pathological conditions (143, 155, 156). All mammalian cells have antioxidant systems to prevent excessive accumulation of ROS that cause oxidative stress leading to disruption of the cellular homeostasis and, eventually, to cell death.

Mitochondrial complexes I and III are the major cellular source of reactive oxygen species. Complex II also produces ROS in both forward and reverse electron flow (157, 158). Superoxide anion (O_2_^-^) is generated during oxidative phosphorylation when leaky electrons partially reduce molecular oxygen. Superoxide is dismutated to hydrogen peroxide (H_2_O_2_) in mitochondria by superoxide dismutase 2. H_2_O_2_ then diffuses to the cytosol or is reduced to water by the thioredoxin-dependent peroxyredoxin system and the glutathione-dependent glutathion peroxidase in the mitochondria (159). In the cytosol, several enzymes also produce H_2_O_2_, including NAD(P)H oxidase (NOX) and cyclooxygenase (COX). Elegant studies from Martin Brand that have identified the contribution of specific mitochondrial sites to ROS production (160), also showed that the mitochondrial site of H_2_O_2_ production depends on which type of respiratory substrates are available (143, 161, 162).

#### 3.3.1 Fluorophores commonly used to determine mitochondrial ROS

There are fluorescent probes available to image both O_2_^-^ and H_2_O_2_ in live cells and mitochondria (143). CellRox Green dye binds to DNA after being oxidized by ROS and therefore shows mostly mitochondrial localization (144, 145, 163) (**Figure 3**). Mitochondrial O_2_^-^ can be imaged using MitoSox Red (**Figure 3**) which is a derivative of hydroethidine, or MitoSox Green mitochondrial superoxide indicator. Although these dyes specifically accumulate in the mitochondria, at high concentrations they also accumulate in the nucleus (146, 164). Thus, MitoSox dyes should be titrated for each cell type. Also, because the accumulation of MitoSox dyes in mitochondria is membrane potential driven, MitoSox fluorescence should be measured together with ΔΨm indicators as additional controls (142).

For imaging H_2_O_2_ in living cells, one of the most widely used fluorescent probe is carboxymethyl 2’,7’-dichlorodihydrofluorescein diacetate (CM-H_2_DCFDA) (**Figure 3**). This probe is not mitochondria specific and can detect more than one type of ROS (Molecular Probes Handbook Table 18.1). Since H_2_O_2_ diffuses from mitochondria to the cytosol and cytosolic NOX is also a significant contributor to its production, it can be challenging to show how much H_2_O_2_ originates from mitochondria. Some investigators use a co-localization approach and overlay ROS signals with membrane potential independent mitochondrial staining (MitoTracker Green FM for example) (142). MitoTracker Deep Red FM can also be used to localize mitochondria (141). To overcome this problem, inhibitors of specific sites of mitochondrial ROS production and mitochondrially targeted antioxidants have been developed. However, compounds used for this purpose should not interfere with mitochondrial function including the activity of the ETC (140, 165). Mitochondria-targeted antioxidants include Mito-Q (ubiquinone attached to a triphenylphosphonium cation) (166), MitoVit-E (vitamin E attached to a triphenylphosphonium cation) and Mito-Tempo (nitroxide conjugated to a triphenylphosphonium cation) (167). The specific proton leak suppressors S1QELs and S1QEL3s have been developed to specifically inhibit the site of production of O_2_^-/^H_2_O_2_ production (168). However, similarly to MitoSox, mitochondria targeted antioxidants have a lipophilic triphenylphosphonium cation (TPP^+^) as delivery conjugate and their accumulation in mitochondria also depends on ΔΨm. Currently, there is no ideal method to quantitatively determine ROS (164).

MitoTracker Orange CM-H_2_TMRos and MitoTracker Red CM-H_2_XRos are derivatives of dihydrotetramethyl rosamine and dihydro-X-rosamine, respectively, that are also utilized for measuring mitochondrial ROS in general. These dyes are not specific for O_2_^-^ and accumulation in the mitochondria is ΔΨm dependent. Because of these limitations, data obtained with these dyes may be difficult to interpret (169).

#### 3.3.2 Genetically encoded ROS sensors

In addition to the above described dyes to measure H_2_O_2,_ there are genetically encoded sensors available including the various Hyper sensors (Hyper1-3, HyperRed) (170–172), Ro-gfp (173), Rogfp2-Orp1 and Grx1-Rogfp2 (117). MitoHyPer, a ratiometric sensor developed to specifically determine mitochondrial H_2_O_2_, can be utilized with its cytosolic counterpart to image cell compartment specific H_2_O_2_ distribution (174). Further, there are also mitochondrial inter- membrane space and mitochondrial matrix-targeted Hyper sensors available (175).

Similar to ΔΨm probes, fluorescent probes and sensors detecting ROS were designed and characterized for single photon application therefore, the authors recommend the use of widefield and confocal microscopy.

### 3.4 ATP

ATP is produced in mitochondria through oxidative phosphorylation and in the cytosol by glycolysis. Imaging ATP using light microscopy is quite challenging. If properly excited with a wavelength of ~259 nm, the emission maxima of ~370 nm is easily masked by autofluorescent tryptophan-containing proteins. Therefore, imaging endogenous ATP or ADP/ATP ratio is not feasible in living cells without the help of sensors.

#### 3.4.1 Genetically encoded ATP sensors and dyes

Genetically targeted luciferase-based probes have been used successfully to measure ATP in live cells and whole animals (176–178). The disadvantage of these probes is that substrate addition is needed during experiments. For fluorescent imaging several sensors have been developed. FRET-based sensors are mostly used for microplate assays (179–181), but there are FRET-based sensor, including ATeam measure ATP levels in the mitochondrial matrix (75). The rhodamine-based sensor RSL^+^ has been used to measure mitochondrial ATP in fibroblasts (182). A different technology, a mitochondria-targeted fluorescence DNA aptamer sensor was also introduced recently to measure mitochondrial ATP in live cells (183). The activity of this sensor is photo-regulated, it only fluoresces after a photo-cleavable linker is removed from the molecule (at 365 nm), and mitochondria targeting is achieved by using DQAsomes, which are highly positive charged liposome-like vesicles. This sensor has fast response time and high signal intensity. Similarly, a ratiometric (FRET-based) fluorescent DNA nanostructure was also reported for imaging of mitochondrial ATP in live cells (76). Near-infrared mitochondria-targeted ATP-binding probe (NIR-A) can provide opportunity for multiplexing and simultaneously measure several mitochondrial parameters (184).

Dyes sensing ATP/ADP ratios include Perceval (185) and PercevalHR (186)), which can be used for single and two-photon microscopy. These probes are not mitochondria targeted, therefore additional subcellular labeling is necessary to localize the signal into the mitochondria. Since some of the ATP sensors are pH sensitive, imaging media and buffers need to be tightly controlled. Also, the use of pH sensitive probes concomitantly with ATP sensors are required to validate the data obtained with ATP sensors.

The above-described fluorescent mitochondrial ATP sensors were developed and validated for single photon imaging. Further, the ADP/ATP sensing non-mitochondria specific dyes need multiplexing with mitochondria-specific dyes to confirm ROS localization which is more feasible using single photon microscopy. Therefore, the authors recommend the use of widefield and confocal microscopy for the detection of mitochondrial ATP.

## 4 Authors perspective and concluding remarks

Overall, there is a growing interest in imaging mitochondrial metabolism and to validate metabolic imaging as a tool to follow carcinogenesis, evaluate tumor therapy and to distinguish between cancerous and healthy tissue (64). The capability of applying these techniques to intraoperative histopathology at subcellular resolution (65) may revolutionize cancer therapy in the future (66, 67). Light microscopy in the different imaging modalities described here became an essential tool to study mitochondrial metabolism in cancer. Widefield, and single photon/multiphoton confocal microscopy have been proved extensively to be excellent and, sometimes, irreplaceable tools to study mitochondrial metabolism in live cells. The capability of determining NADH, NAD, FAD, ΔΨm, ROS and ATP in real time allows to study the progression of changes in mitochondrial metabolism under physiological, pathological or pharmacologically induced conditions. At present, any research on cancer metabolism that, directly or indirectly, may relate to modifications of mitochondrial function, should include some or all the image applications described in this review. Here, we introduced several tools, microscope modalities, use of autofluorescence, fluorescence sensors and dyes to study mitochondrial metabolism highlighting their advantages and disadvantages.

Important problems that still need to be addressed to improve metabolic imaging and probe development include specificity, toxicity, and molecular targeting. Loading of a dye or overexpression of a molecule, can disturb cellular metabolism. Therefore, it is of high relevance to develop highly specific mitochondrial dyes and sensors that can be used at low concentration. Examples of these are: a) Fluorescent dyes that could distinguish between NADH and NADPH; b) Mitochondria targeted non-potentiometric dyes that could be used to colocalize metabolic signals to the organelle. Currently this selection is very limited; c) Low toxicity potentiometric dyes which can be used for multiplexing; d) Specific, ΔΨm *insensitive* ROS dyes labelling only one specific ROS molecule; and e) Multiphoton probes for multiplexing metabolic imaging with autofluorescence. Another limiting factor for the use of state-of-the art microscopy techniques like multiphoton microscopy, FLIM and several super-resolution modalities are the high price and lack of technical expertise. However, studies to determine relationships between metabolic activity and mitochondrial dynamics (out of the scope of our review), assembly of molecular complexes of the respiratory chain and the ATP synthase, as well as colocalization and potential interaction of signaling molecules in mitochondria could greatly benefit of super-resolution capability.

In general, education in imaging and available technologies together with decreased pricing could increase the use of microscopy for future cancer metabolism studies and ultimately cancer diagnostics.

## Author contributions

MG and EM retrieved concerned literature, wrote and edited manuscript, designed and prepared figures. Both authors contributed to the article and approved the submitted version.
